# Temporal Histopathological and Immunohistochemical Analysis of Cutaneous Injury and Wound Repair in Hairless Guinea Pigs Following Sulfur Mustard Exposure

**DOI:** 10.46889/jdr.2026.7301

**Published:** 2026-09-02

**Authors:** Kevin Ozkuyumcu, Daniel Weinstock, Peihong Zhou, Claire R Croutch, Jeffrey D Laskin, Laurie B Joseph

**Affiliations:** 1 Department of Pharmacology and Toxicology, Ernest Mario School of Pharmacy, Rutgers University, Piscataway, NJ, USA; 2 MRIGlobal, Kansas City, MO, USA; 3 Department of Environmental and Occupational Health and Justice, School of Public Health, Rutgers University, Piscataway, NJ, USA

**Keywords:** Hairless Guinea Pig, Keratinocytes, Microblisters, Vesicants, Wound Healing

## Abstract

Sulfur Mustard (SM) is a potent vesicant that causes severe cutaneous injury characterized by inflammation, blistering and long-term impairment of skin barrier function. Although the clinical manifestations of SM exposure are well documented, the temporal processes governing epidermal injury and repair remain incompletely defined. In this study, hairless guinea pigs, a translational model with close structural similarity to human skin, were used to characterize acute injury and subsequent wound healing following SM exposure. Within 2 days of exposure, SM induced extensive epidermal and dermal appendage necrosis, dermo-epidermal clefts, superficial dermal vascular injury, inflammatory cell infiltration, marked disruption of elastic fibers and edema in the hypodermis. These changes were accompanied by decreased expression of markers of keratinocyte proliferation, adhesion and differentiation, including Proliferating Cell Nuclear Antigen (PCNA), E-cadherin, keratins and loricrin. By 20 days post-exposure, a hyperplastic neoepidermis was evident together with acanthosis and perinuclear vacuolization of the epithelium. Partial restoration of E-cadherin expression, along with increased expression of PCNA, keratins and loricrin, was consistent with ongoing epithelial repair. However, dermal remodeling remained incomplete, with persistent inflammatory cells and abnormalities in collagen and elastic fiber organization. By 66 days post-exposure, epidermal thickness and markers of proliferation and differentiation approached control skin; however, loss of adnexal structures and the development of a compact, collagen-dense superficial dermis indicated scar-like remodeling. These findings suggest that SM-induced skin injury is followed by prolonged and dysregulated tissue repair, resulting in restoration of epidermal architecture lacking adnexal structures and collagen remodeling in the superficial dermis that may compromise normal skin structure and function.

## Introduction

Sulfur Mustard (SM; bis(2-chloroethyl) sulfide) is a highly reactive, bifunctional alkylating agent used in chemical warfare [1-3]. Due to its lipophilic nature, SM readily penetrates the skin barrier, where it induces cellular injury, inflammation and tissue destruction [4]. Clinically, cutaneous injury progresses from delayed-onset erythema to edema with inflammatory cell infiltration, followed by the formation of large fluid-filled blisters [5,6]. Blister rupture results in necrotic ulcerative lesions that become covered by a scab during healing [7]. Despite wound closure, long-term sequelae, including hypo- or hyperpigmentation, chronic skin abnormalities and scarring are common, reflecting prolonged and often incomplete tissue repair [8-12].

The hairless guinea pig is a well-established model for investigating SM-induced skin injury because its cutaneous architecture closely resembles that of human [13-15]. The epidermis is relatively thick and well stratified, with a compact stratum corneum, while the dermis contains abundant collagen bundles, elastic fibers, fibroblasts and resident immune cells [16,17]. In addition, the dermal microvasculature, including superficial vascular plexuses and vertically oriented capillary loops, closely parallels that of human skin [18,19], making this model particularly suitable for studies of vesicant-induced injury and repair.

Although the acute pathological effects of SM have been extensively described, the temporal sequence of epidermal degeneration, dermal remodeling and wound repair remains incompletely understood. In particular, the cellular and structural changes that accompany the transition from acute injury to tissue regeneration and chronic remodeling have not been fully characterized. A better understanding of these processes is needed to identify mechanisms that contribute to delayed healing and persistent tissue damage and to support the development of effective medical countermeasures.

The present study characterizes the temporal histopathological changes associated with SM-induced skin injury in the hairless guinea pig over a 66-day period following a single topical exposure. Histochemical and immunohistochemical analyses were used to evaluate epidermal proliferation and differentiation, cell adhesion, inflammatory responses and remodeling of the dermal extracellular matrix during acute injury and subsequent wound repair. Although descriptive in nature, these findings provide insight into the progression of SM-induced cutaneous pathology and identify structural and molecular changes that may inform future mechanistic studies and the development of therapies to improve wound healing following vesicant exposure.

## Materials and Methods

### Animals and Treatments

Hartley hairless guinea pigs (6-8 weeks old) were obtained from Charles River Laboratories (Wilmington, MA). All SM exposures were performed at MRIGlobal (Kansas City, MO) in an Association for Assessment and Accreditation of Laboratory Animal Care accredited facility under approved Institutional Animal Care and Use Committee protocols following previously published methods [20]. Animal studies were performed in compliance with Association for Assessment and Accreditation of Laboratory Animal Care International guidelines. All experiments were approved by the Institutional Animal Care and Use Committee at MRIGlobal (Protocol # 110858). All animals were given humane treatment and measures were taken to minimize animal suffering during these studies. Animals were randomly assigned to treatment groups before exposures to SM. For vapor exposures, animals were restrained and 14 mm diameter vapor caps secured to the dorsal surface using double-sided adhesive tape [21].

### Sulfur Mustard Exposure, Debridement and Tissue Collection

To generate a saturated SM vapor, 10 μL of SM was applied to a filter placed inside each cap. The cap was then placed on a glass slide for 5 min to allow vapor equilibration before application to the skin [22]. As previously reported, the equilibrium vapor pressure of SM is 0.090 mmHg at 30°C, corresponding to an estimated vapor concentration of approximately 1.4 mg/L [23]. Hairless guinea pig skin was exposed to SM vapor or control vapor for 20 min using this vapor-cap model as previously described [20]. Beginning 2 days after exposure and continuing once daily for the following 4 days, both SM-exposed and control sites underwent standardized wound debridement using wet-to-wet saline gauze dressings. This procedure was performed on all animals to ensure consistent wound care and minimize potential bias associated with wound management. Animals were euthanized 2, 20 or 66 days after exposure. Full-thickness 7-mm punch biopsies were collected from the dorsal skin at the interface between SM-exposed and adjacent untreated skin. Tissue samples were fixed in paraformaldehyde-sucrose in Phosphate-Buffered Saline (PBS), processed routinely and embedded in paraffin. Sections (6 μm thick) were prepared by the Histopathology Core Facility at Rutgers University.

### Histopathological Evaluation

For histopathological evaluation, tissue sections were deparaffinized and stained with Hematoxylin and Eosin (H&E) to assess overall tissue architecture and cellular morphology or with Masson's trichrome to evaluate collagen organization and dermal remodeling. Changes in dermal elastic fibers were assessed using an orcein-based staining kit (StatLab, McKinney, TX). Digital images were acquired at 200× magnification using an Olympus VS120 Virtual Microscopy System and analyzed with OlyVIA software (Olympus, Center Valley, PA).

### Epidermal Thickness and Statistics

To quantify epidermal thickness, H&E-stained sections from SM-exposed and control skin were scanned at 200× magnification using an Olympus VS120 Virtual Microscopy System. For each tissue section, epidermal thickness was measured at 20 evenly distributed inter-rete regions by drawing perpendicular lines from the basement membrane to the base of the stratum corneum, as previously described [24,25]. The mean epidermal thickness for each biopsy was calculated and used for statistical analysis. Data were analyzed by one-way analysis of variance (ANOVA) followed by Dunnett's multiple-comparison test. Results are presented as the mean ± SEM (n = 4 biopsies per time point, representing two biopsies from each of two animals). Differences were considered statistically significant at p < 0.05.

### Immunohistochemistry

Tissue sections were deparaffinized and blocked for 2 h at room temperature with either normal goat or horse serum (Thermo Fisher Scientific, Waltham, MA) to reduce nonspecific antibody binding. Sections were then incubated overnight at 4°C with primary antibodies against keratin 5 (1:20,000; Abcam, Waltham, MA), keratin 10 (1:500; Abcam), keratin 17 (1:2,000; Novus Biologicals, Centennial, CO), E-cadherin (1:150; Abcam), Proliferating Cell Nuclear Antigen (PCNA; 1:1,000; MilliporeSigma, St. Louis, MO) or loricrin (1:400; Abcam). After washing with PBS, sections were incubated for 30 min at room temperature with the appropriate biotinylated secondary antibody (goat anti-rabbit or horse anti-mouse). Immunoreactivity was visualized using a 3,3′-diaminobenzidine (DAB) peroxidase substrate kit (Vector Laboratories, Newark, CA) and sections were counterstained with Mayer's hematoxylin (Electron Microscopy Sciences, Hatfield, PA). Digital images were acquired at 200× magnification using an Olympus VS120 Virtual Microscopy System and analyzed with OlyVIA software (Olympus, Center Valley, PA).

## Results

### Structural Changes in Hairless Guinea Pig Skin After SM Exposure

In control animals, the epidermis exhibited four distinct layers including a well-developed compact stratum corneum, a stratum granulosum composed of flattened keratinocytes, a stratum spinosum containing polyhedral epithelial cells and a highly proliferative stratum basale (Fig. 1-3). The dermis displayed a well-organized, interwoven collagen network with interspersed hair follicles and follicular infundibula accompanied by adjacent sebaceous glands; hair follicles were devoid of hair shafts. Scattered resident leukocytes and fibrocytes were present throughout the dermis. Elastic fibers were dense and highly branched within the superficial dermis. Approaching the basement membrane, elastic fibers adopted a perpendicular, thread-like orientation, whereas deeper dermal fibers exhibited a mixed orientation with multiple branch points (Fig. 4).

By 2 days post-exposure, SM-induced skin lesions were characterized by deep ulcers with full-thickness epidermal necrosis. The necrotic epidermis overlaid a dermo-epidermal cleft, beneath which a layer of necrotic and degenerate heterophils (the functional equivalent of neutrophils in guinea pigs) was present, with few accompanying mononuclear cells (Fig. 2,3). Lesions at the ulcer margins were less severe and exhibited pan-epidermal degeneration with multifocal basal keratinocyte necrosis. Epidermal thickness did not differ significantly from control skin at this time (Fig. 5). The superficial dermis was mildly edematous and contained moderate perivascular inflammatory infiltrates surrounding blood vessels with vascular injury. Evidence of vascular injury was further supported by extravasation of red blood cells 2 days following SM exposure (Fig. 2,3). Elastic fiber architecture was altered, with reduced branching and a predominance of fibers oriented parallel to the basement membrane; few fibers extended perpendicular to the Dermal-Epidermal Junction (DEJ) (Fig. 4). Collagen bundles in the superficial dermis were fragmented, separated by expanded perivascular and interstitial space and exhibited altered stain intensity with Masson’s Trichrome (Fig. 1,3).

By 20 days post-exposure, re-epithelialization resulted in the formation of a neoepidermis exhibiting moderate acanthosis with a significant increase in thickness compared to control skin (103.9 ± 7.6 μm vs. 46.2 ± 6.5 μm, respectively; p ≤ 0.05) (Fig. 1-3,5).

At this time point (16 days post-debridement), the stratum corneum was well developed and compact. Perinuclear vacuolization was evident within keratinocytes of the stratum spinosum and granulosum, which persisted for at least 66 days post-SM exposure. Inflammatory cell infiltration and overall cellularity within the superficial dermis were reduced by 20 days post-SM compared with 2-day post-SM skin, although mild dermal edema remained evident at the DEJ (Fig. 1-3). Elastic fibers in the superficial dermis decreased in number and appeared fragmented and agglomerated, with reduced branching (Fig. 4). Denatured collagen in the superficial dermis appeared homogeneous and glassy with loss of fibrillar and bundle architecture. Edema in the hypodermis (subcutis) had resolved (Fig. 1,3).

By 66 days post-SM exposure, full-thickness epithelial regeneration of the epidermis had occurred with mild acanthosis and hyperkeratosis while stratum corneum approached control skin morphology. Epidermal thickness was similar to that of control skin at this time point (Fig. 5). Microblisters were absent, with no evidence of dermo-epidermal cleft formation and a clear demarcation between the superficial and deep dermis was apparent. The superficial dermis consisted of lighter staining, dense, highly compacted collagen fibers oriented parallel to the DEJ and was devoid of hair follicles and other adnexal structures, a pattern consistent with human scar histology (Fig. 1-3) [26, 27]. In contrast, the deep dermis contained interwoven collagen fibers approximating those observed in control skin, although secondary appendages remained absent (Fig. 1,3). This superficial-deep dermal boundary was further highlighted by elastic fiber staining, which revealed an absence of elastic fibers in the superficial dermis and their presence only in the deep dermis, where they exhibited a predominantly horizontal orientation (Fig. 4).

### SM-Induced Changes on Markers of Keratinocyte Growth and Development in Hairless Guinea Pig Skin

PCNA, a marker of cellular proliferation, was localized to the nuclei of basal keratinocytes and, to a lesser extent, suprabasal cells in control epidermis, consistent with normal epidermal turnover and baseline epidermal thickness (Fig. 6) [28]. Expression of PCNA was also observed in epidermal cells of the outer root sheath of hair follicles and dermal interstitial cells in control skin. At 2 days post-SM exposure, PCNA expression was minimal within the necrotic epidermis and absent along the basal layer, paralleling the lack of change in overall epidermal thickness at this early injury stage. Little to no PCNA staining was detected in cells of the superficial dermis, including immune cells, fibroblasts or dermal adnexa.

By 20 days post-SM, during active re-epithelialization and formation of a hyperplastic neoepidermis, PCNA expression was markedly increased in the nuclei of keratinocytes within the stratum basale and stratum spinosum, consistent with the significant increase in epidermal thickness observed at this stage. PCNA-positive immune cells and fibroblasts were also evident within the dermis. At 66 days post-SM, as the neoepidermis underwent remodeling, PCNA expression was predominantly restricted to contiguous basal keratinocytes, with minimal suprabasal staining, consistent with normalization of epidermal thickness toward control levels. PCNA-positive immune cells and fibroblasts persisted within the dermis at this later stage.

E-cadherin, a cell-cell adhesion protein critical for maintaining the epidermal architecture was uniformly expressed throughout the viable epidermis in control skin (Fig. 7), with membrane localization also evident in hair follicle cells and sebocytes [29-31]. At 2 days post-SM, E-cadherin was markedly reduced in both the secondary appendages and the epidermis, where it was localized predominantly to the suprabasal layer. By 20 days post-SM, during active re-epithelialization and formation of a hyperplastic neoepidermis, E-cadherin expression in the epidermis approached control levels, coinciding with increased PCNA expression and epidermal thickening. At 66 days post-SM, following epidermal remodeling, E-cadherin expression remained similar to control, consistent with the restoration of basal keratinocyte proliferation patterns and normalization of epidermal architecture.

### SM-Induced Changes on Markers of Keratinocyte Growth and Differentiation in Hairless Guinea Pig Skin

In the epidermis and dermal appendages, keratins provide structural support that confers mechanical resilience and contributes to barrier formation. They also regulate key cellular processes, including keratinocyte proliferation and differentiation, which are essential for epidermal repair following injury. Accordingly, the layer-specific expression of keratins permits evaluation of basal layer integrity, stress-associated activation and suprabasal differentiation during cutaneous injury and wound healing [32,33].

In hairless guinea pig skin, keratin 5, a basal keratin required for anchoring keratinocytes to the basal lamina, was primarily expressed in basal keratinocytes of control epidermis and in the outer root sheath of hair follicles (Fig. 8). At 2 days post-SM, keratin 5 expression was reduced in basal keratinocytes and in the outer root sheath, consistent with acute basal layer injury. By 20 and 66 days post-SM, keratin 5 expression in basal keratinocytes had largely returned to control levels; however, secondary appendages, including keratin 5-expressing hair follicles, were absent at 66 days.

Keratin 17, a stress- and wound-associated keratin that supports keratinocyte activation, proliferation and migration, was detected in basal keratinocytes of control skin and in epidermal cells of the outer root sheath of hair follicles (Fig. 9) [22,34,35]. At 2 days post-SM, keratin 17 expression was reduced in the stratum basale, with no effect on expression in hair follicles. During active re-epithelialization at 20 days post-SM, keratin 17 was diffusely expressed throughout the viable epidermis, with prominent basal localization, consistent with activation of keratinocytes within the neoepidermis. By 66 days post-SM, keratin 17 expression returned to a control-like pattern restricted to the basal layer; however, keratin 17 expressing hair follicles were absent due to loss of secondary appendages.

Keratin 10, a suprabasal keratin and marker of early keratinocyte differentiation, was not detected in the basal layer of guinea pig skin but was expressed in suprabasal layers of both control and SM-exposed epidermis (Fig. 10) [22,36]. Keratin 10 expression was upregulated at 20 days post-SM, consistent with enhanced suprabasal differentiation during formation of the hyperplastic neoepidermis and remained restricted to suprabasal layers at later time points.

Loricrin, a structural protein essential for stratum corneum barrier integrity and a marker of terminal differentiation and keratinocyte cornification, was constitutively expressed at the base of the stratum corneum in control skin (Fig. 11) [37,38]. At 2 days post-SM, loricrin expression was downregulated and localized to the stratum granulosum, indicating disruption of terminal differentiation following acute injury. By 20 days post-SM, loricrin was upregulated along the base of the stratum corneum with diffuse expression within the hyperplastic epidermis, consistent with re-establishment of cornification during re-epithelialization. At 66 days post-SM, loricrin expression approached control levels, with contiguous staining at the base of the stratum corneum, reflecting maturation of the neoepidermis and partial restoration of barrier architecture.

## Discussion

In this study, we used the hairless guinea pig to characterize the temporal progression of cutaneous injury and repair following Sulfur Mustard (SM) exposure. This model was selected because its skin closely resembles human skin in both epidermal organization and dermal architecture, making it well suited for studies of vesicant-induced injury [15]. The stratified epidermis, well-developed stratum corneum, collagen-rich dermis, elastic fiber network and resident stromal and immune cells provide a relevant framework for examining the structural and molecular events that accompany SM-induced tissue damage and wound healing.

Consistent with previous reports, early injury (2 days post-exposure) was characterized by extensive epidermal necrosis, dermo-epidermal separation, vascular injury and acute inflammatory cell infiltration [21,39-41]. These pathological changes were accompanied by marked reductions in markers of keratinocyte proliferation, adhesion and differentiation, indicating widespread disruption of epidermal homeostasis. By 20 days post-exposure, re-epithelialization resulted in formation of a hyperplastic neoepidermis with significantly increased epidermal thickness, persistent keratinocyte vacuolization and altered expression of keratinocyte differentiation markers. Although these changes are consistent with active tissue repair, they also indicate that normal epidermal architecture had not yet been fully restored extending previous observations of SM-induced epidermal hyperplasia in experimental models [22,24,42].

Despite substantial restoration of the epidermis by 66 days after exposure, persistent abnormalities remained within the dermis (Fig. 1-3). Collagen fibers in the superficial dermis were densely compacted and aligned parallel to the Dermal-Epidermal Junction, creating a distinct boundary between the superficial and deep dermis characteristic of scar-like remodeling (Fig. 1,3). These changes were accompanied by marked loss of elastic fibers and permanent depletion of pilosebaceous units, consistent with previous studies in the hairless guinea pig [40]. Together, these findings indicate that restoration of epidermal continuity does not equate to complete regeneration of normal skin architecture.

Elastic fiber remodeling was one of the most persistent pathological changes observed following SM exposure. Elastic fibers were fragmented or absent during the acute injury phase and remained markedly reduced in the superficial dermis throughout the study. Because elastic fibers regenerate poorly in adult skin, their persistent loss is likely to contribute to long-term reductions in tissue elasticity and mechanical integrity. Similar alterations have been described in hypertrophic scars and chronic SM-induced skin lesions, suggesting that impaired elastic fiber restoration is a common feature of pathological wound healing [43,44].

Changes in molecular markers further distinguished regenerative repair from complete tissue recovery. PCNA expression demonstrated a transient proliferative response during neoepidermis formation, whereas recovery of E-cadherin expression was consistent with re-establishment of epithelial cell-cell adhesion. However, normalization of these markers occurred despite persistent dermal remodeling and loss of adnexal structures, indicating that restoration of selected epidermal markers alone does not reflect complete tissue repair. Likewise, sustained expression of keratin 17 together with altered expression of keratins 5 and 10 and loricrin suggests prolonged keratinocyte activation and incomplete terminal differentiation, changes commonly associated with chronic wound healing and inflammatory skin disease.

Taken together, these findings demonstrate that SM-induced skin injury progresses through distinct phases of acute tissue destruction, re-epithelialization and prolonged dermal remodeling. Although the epidermis largely regained normal thickness and expression of several differentiation markers by 66 days, persistent abnormalities in the dermal extracellular matrix and permanent loss of skin appendages indicate incomplete regeneration and scar-like repair. These qualitative observations identify histopathological and molecular endpoints that may prove useful for evaluating candidate medical countermeasures designed to improve wound healing following vesicant exposure.

## Conclusion

This study has several limitations. It was designed as an exploratory histopathological investigation and only two biopsies from each of two animals were examined at each time point. Consequently, the findings are primarily descriptive and the limited sample size precludes robust statistical assessment of many pathological changes. In addition, although the hairless guinea pig closely models numerous structural features of human skin, species differences in skin thickness, appendage density, immune responses and wound healing kinetics should be considered when extrapolating these findings to human SM injury. Future studies using larger cohorts and quantitative analyses will be important for confirming the reproducibility of these observations and for defining the mechanisms underlying persistent dermal remodeling following SM exposure.

## Figures and Tables

**Figure 1:**
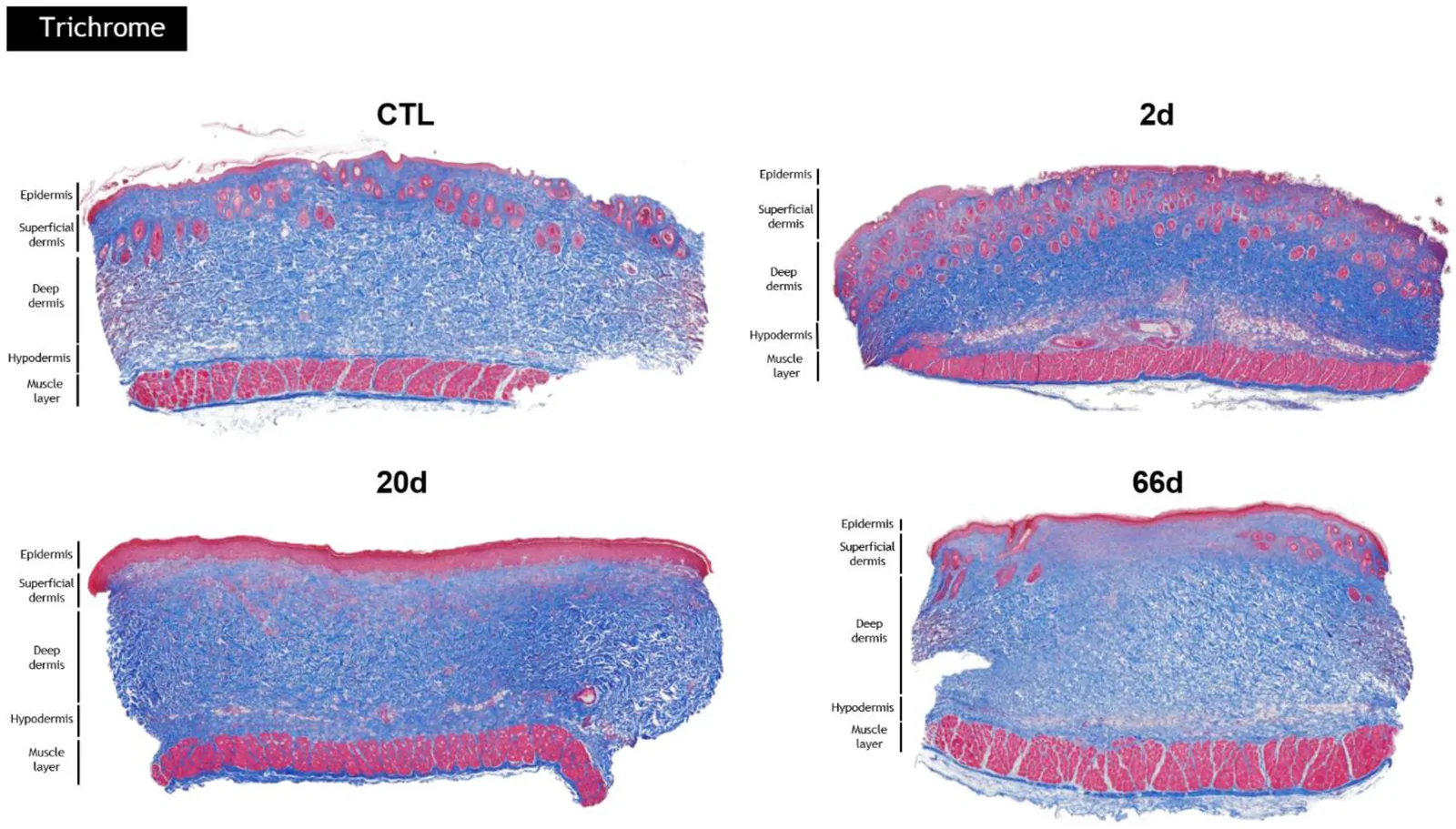
Masson’s trichrome of whole skin sections of hairless guinea pigs following SM exposure. Histological sections, prepared from control (CTL) guinea pig skin and guinea pig skin 2-, 20- and 66-days post SM, were stained with Masson’s trichrome containing hematoxylin which stains nuclei dark blue/black, eosin which stains keratin and cytoplasm red and aniline blue which stains collagen I/III royal blue. Images at 30x magnification are presented. Tissue sections are labeled as epidermis, superficial dermis, deep dermis, hypodermis (subcutis) and muscle layer.

**Figure 2:**
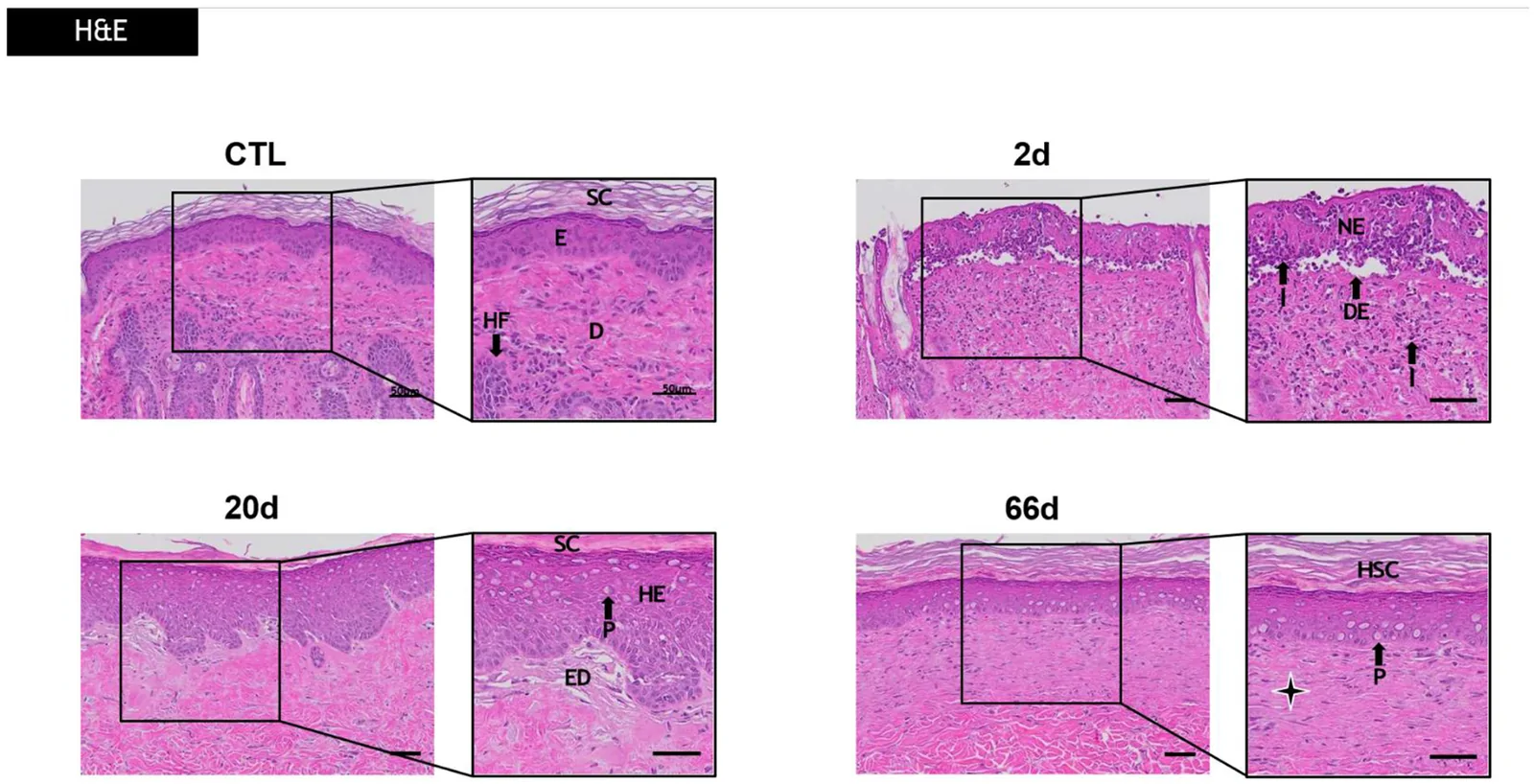
SM-induced histopathological changes in hairless guinea pig skin. Histological sections, prepared from control (CTL) guinea pig skin and guinea pig skin 2-, 20- and 66-days post SM, were stained with H&E. Images at low and high magnifications are presented. D, dermis; DE, dermo-epidermal cleft; E, epidermis; ED, edema; HE, hyperplastic epidermis; HF, hair follicle; HSC, hyperkeratotic stratum corneum; I, inflammatory cell infiltrate; NE, necrotic epidermis; P; perinuclear vacuolization; SC, stratum corneum; Star, horizontal collagen deposition. Original magnification, x200.

**Figure 3:**
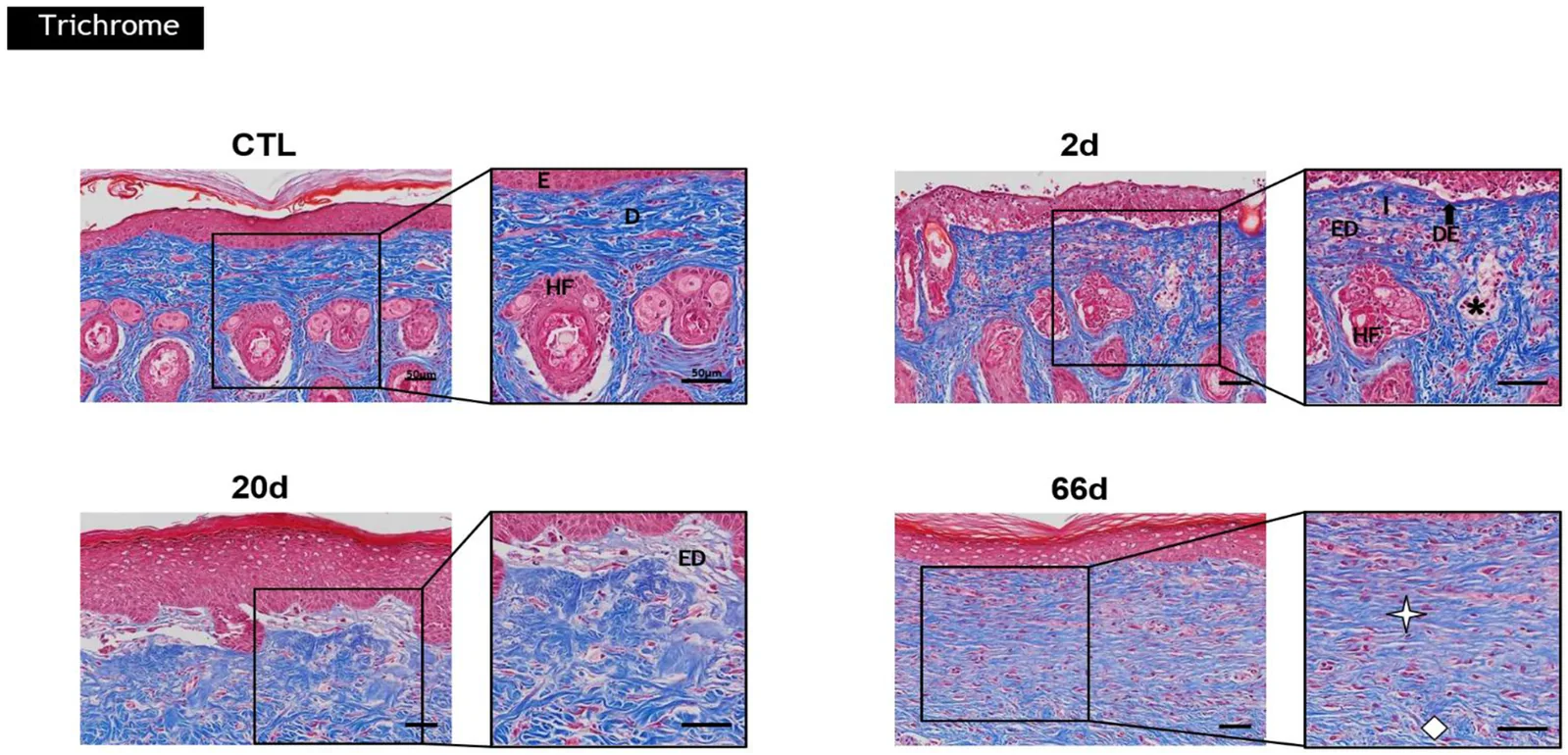
Masson’s trichrome staining of hairless guinea pig skin following SM exposure. Histological sections, prepared from control (CTL) guinea pig skin and guinea pig skin 2-, 20- and 66-days post SM, were stained with Masson’s trichrome containing hematoxylin which stains nuclei dark blue/black, eosin which stains keratin and cytoplasm red and aniline blue which stains collagen I/III royal blue. Images at low and high magnification are presented. D, dermis; DE, dermo-epidermal cleft; E, epidermis; ED, edema; HF, hair follicle; Asterisk, vascular necrosis; Star, horizontal collagen deposition in the superficial dermis; Diamond, deep dermis. Original magnification, x200.

**Figure 4:**
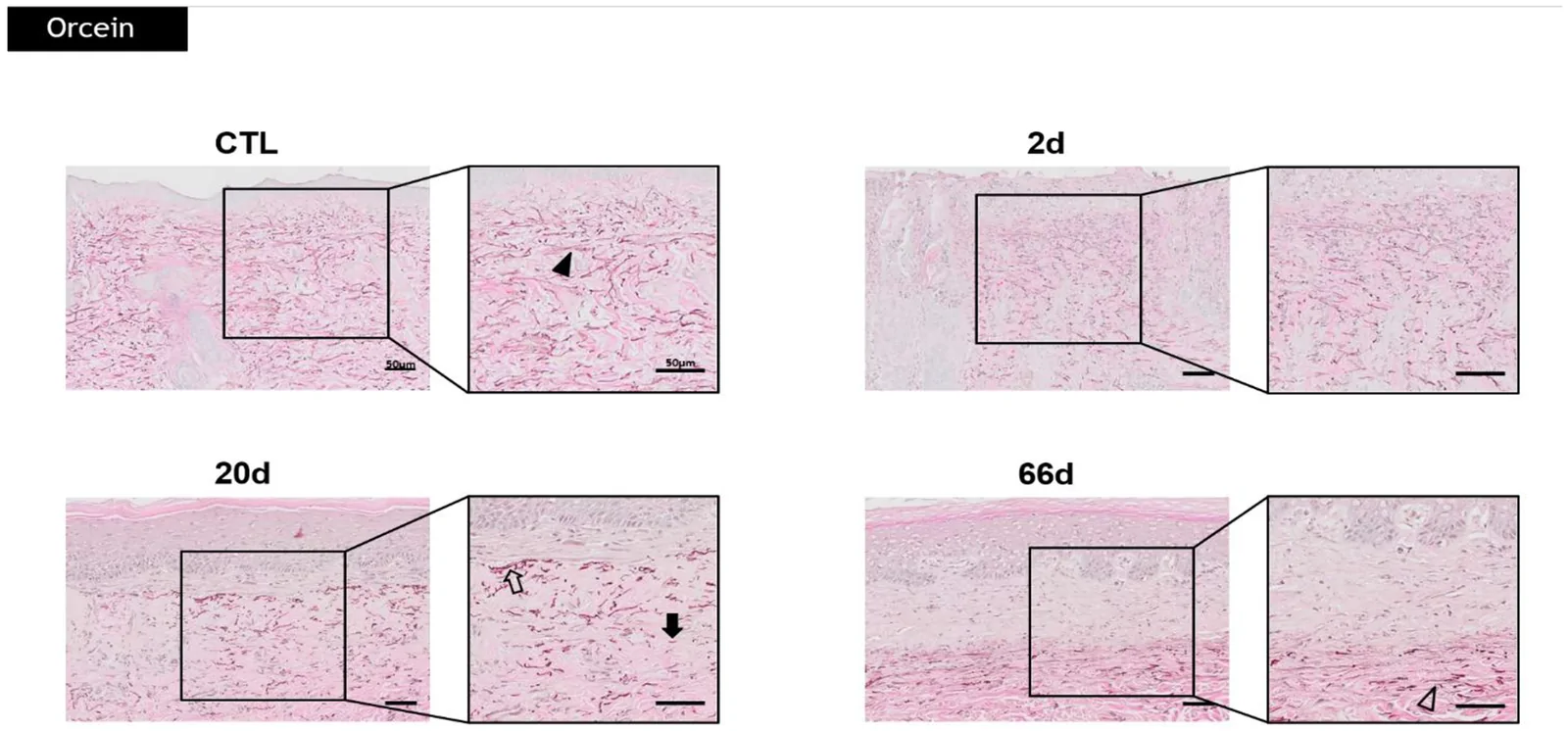
Orcein stain of hairless guinea pig skin following SM exposure. Histological sections, prepared from control (CTL) guinea pig skin and guinea pig skin 2-, 20- and 66-days post SM, were stained with an orcein stain kit containing acid orcein which stains elastic fibers dark purple/brown, dilute Giemsa which stains nuclei deep blue and cytoplasm light blue and eosin Y 0.02% alcohol which stains collagen rose pink. These latter two counterstains provide contrasting nuclear and cytoplasmic coloration. Closed arrow, fragmented elastic fiber; Closed triangle, elastic fiber; Open arrow, agglomerated elastic fiber; Open triangle, horizontally oriented elastic fibers in the deep dermis. Original magnification, x200.

**Figure 5:**
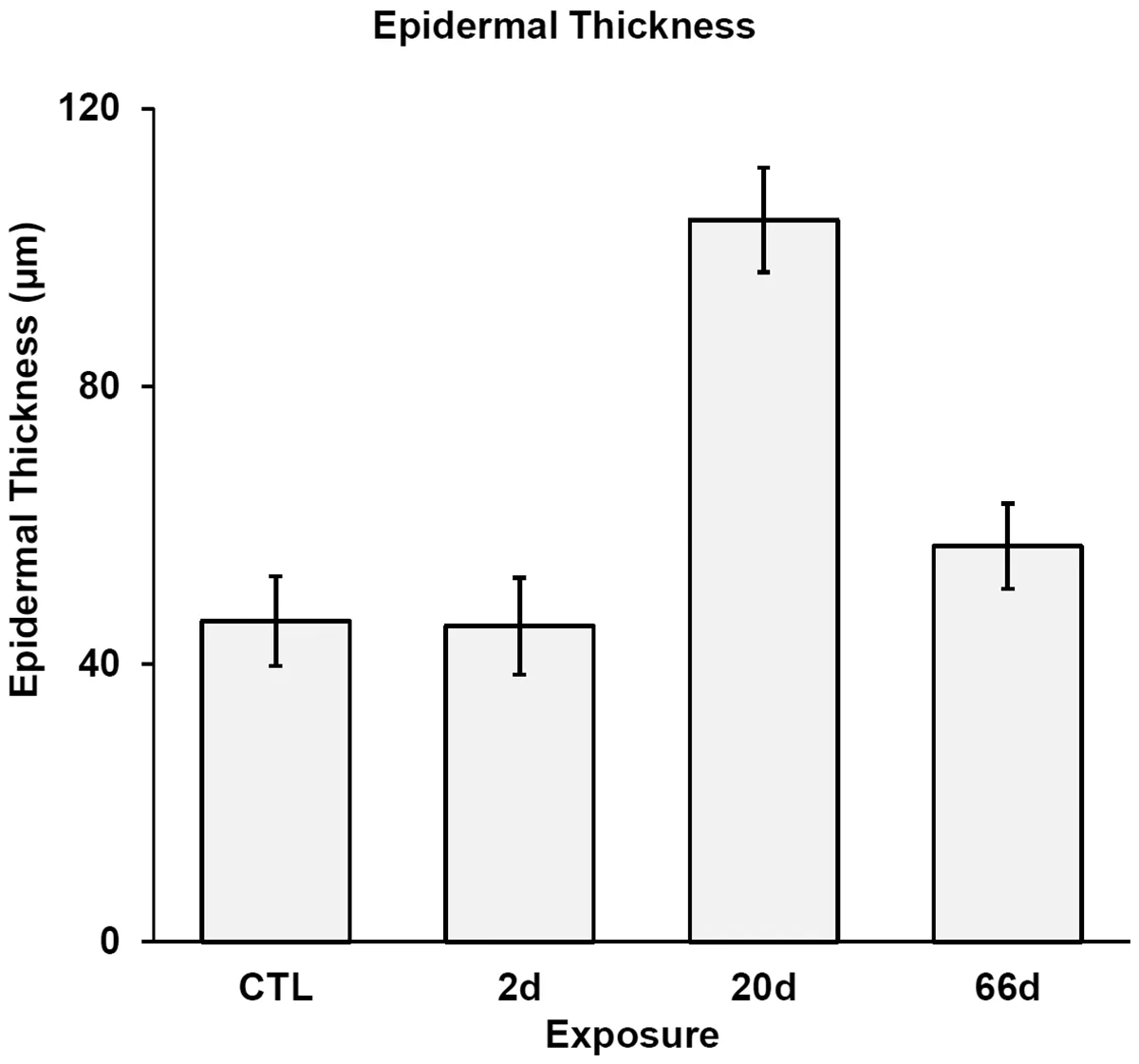
Effect of SM on epidermal inter-rete thickness in hairless guinea pig skin. H&E-stained tissue sections of control (CTL) and SM-exposed skin were scanned and 20 inter-rete measurements from each animal were taken by dropping a perpendicular line from the stratum corneum to the basal layer of the epidermis. CTL: Mean thickness of 46.18 ± 6.45μm. Day 2: Mean thickness of 45.49 ± 6.98μm (p = 0.98). Day 20: A statistically significant increase in epidermal thickness was seen in inter-rete epidermal thickness in 20 days post-SM skin compared to control (103.93 ± 7.55, p < 0.0001). Day 66: Mean thickness was 56.98 ± 6.14μm, which was not significantly different compared to control (p = 0.56). Each bar represents the mean ± SEM, n = 4. *Significantly different from control skin by one-way ANOVA (p ≤ 0.05).

**Figure 6:**
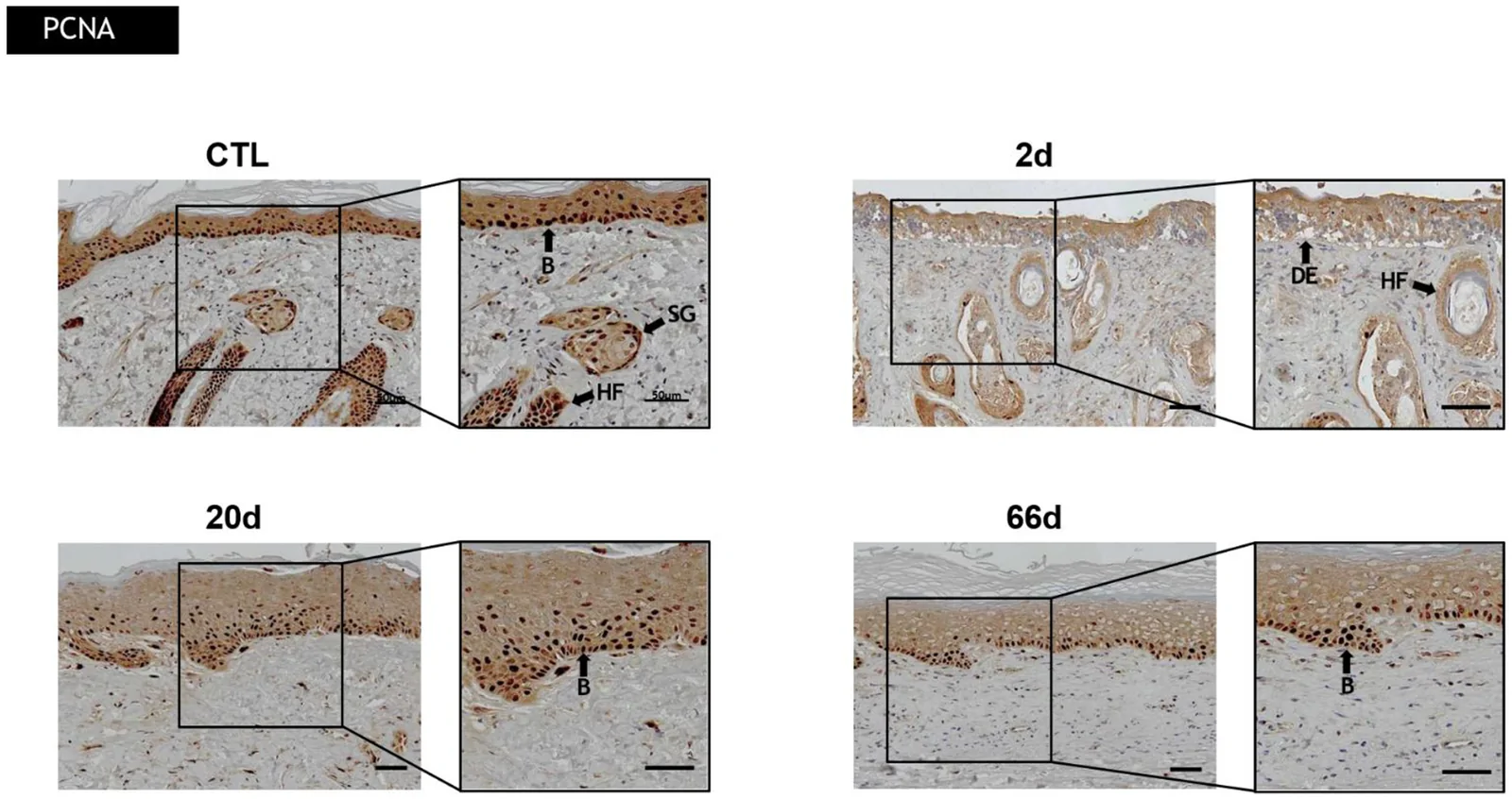
Localization of PCNA in hairless guinea pig skin following SM exposure. Control (CTL) guinea pig skin and 2-, 20-, 66-days post-SM guinea pig skin sections were stained with antibodies against Proliferating Cell Nuclear Antigen (PCNA). Antibody binding was visualized suing Vectastain Elite ABC Kit. Images at low and high magnification are presented. B, basal layer; DE, dermo-epidermal cleft; HF, hair follicle; SG, sebaceous gland. Original Magnification, x200.

**Figure 7:**
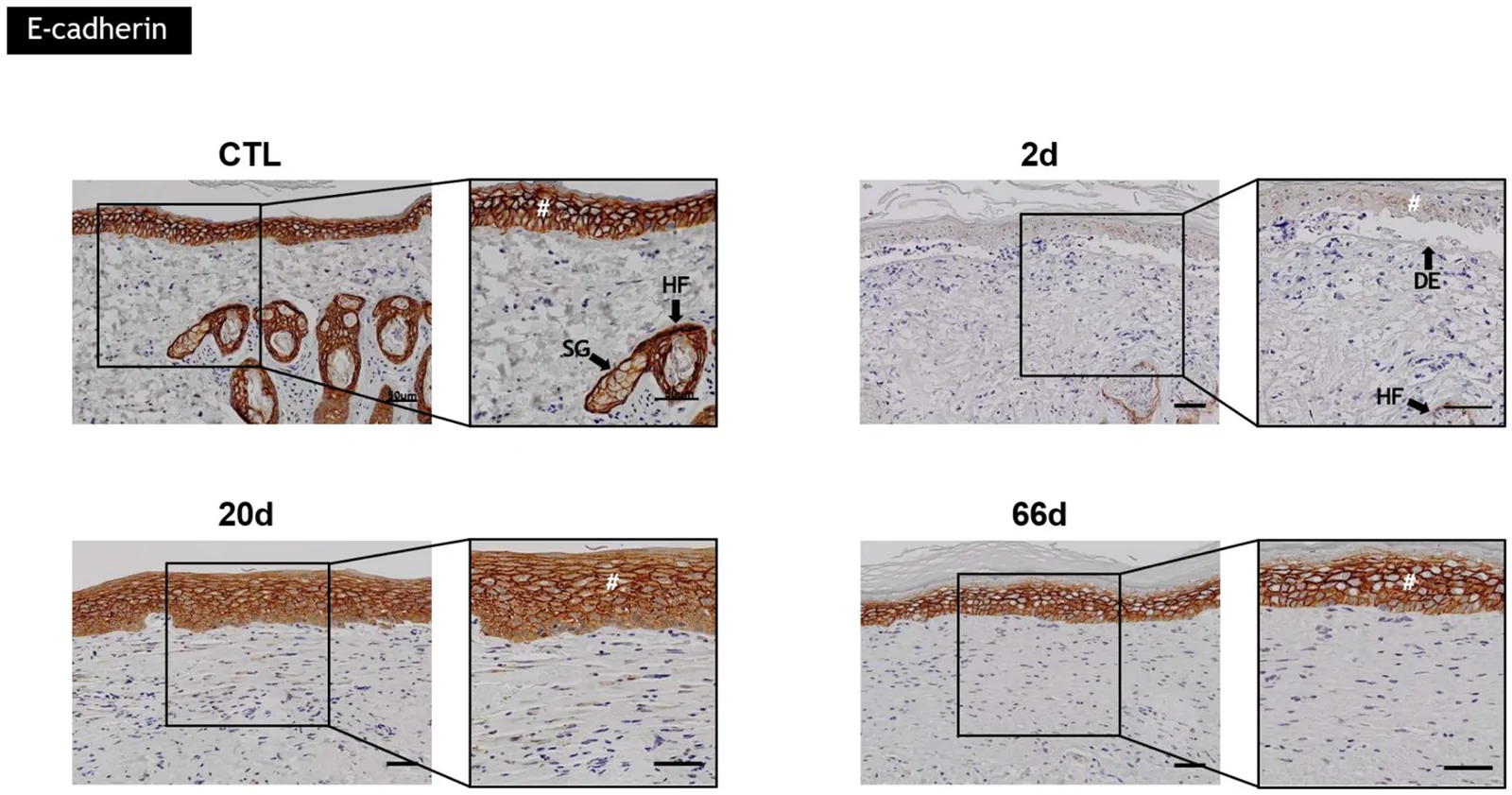
Localization of E-cadherin in hairless guinea pig skin following SM exposure. Control (CTL) guinea pig skin and 2-, 20-, 66-days post-SM guinea pig skin sections were stained with antibodies against E-cadherin. Antibody binding was visualized using a Vectastain Elite ABC kit. Images at low and high magnification are presented. DE, dermo-epidermal cleft; HF, hair follicle; SG, sebaceous gland; Pound sign, suprabasal layer. Original magnification, x200.

**Figure 8:**
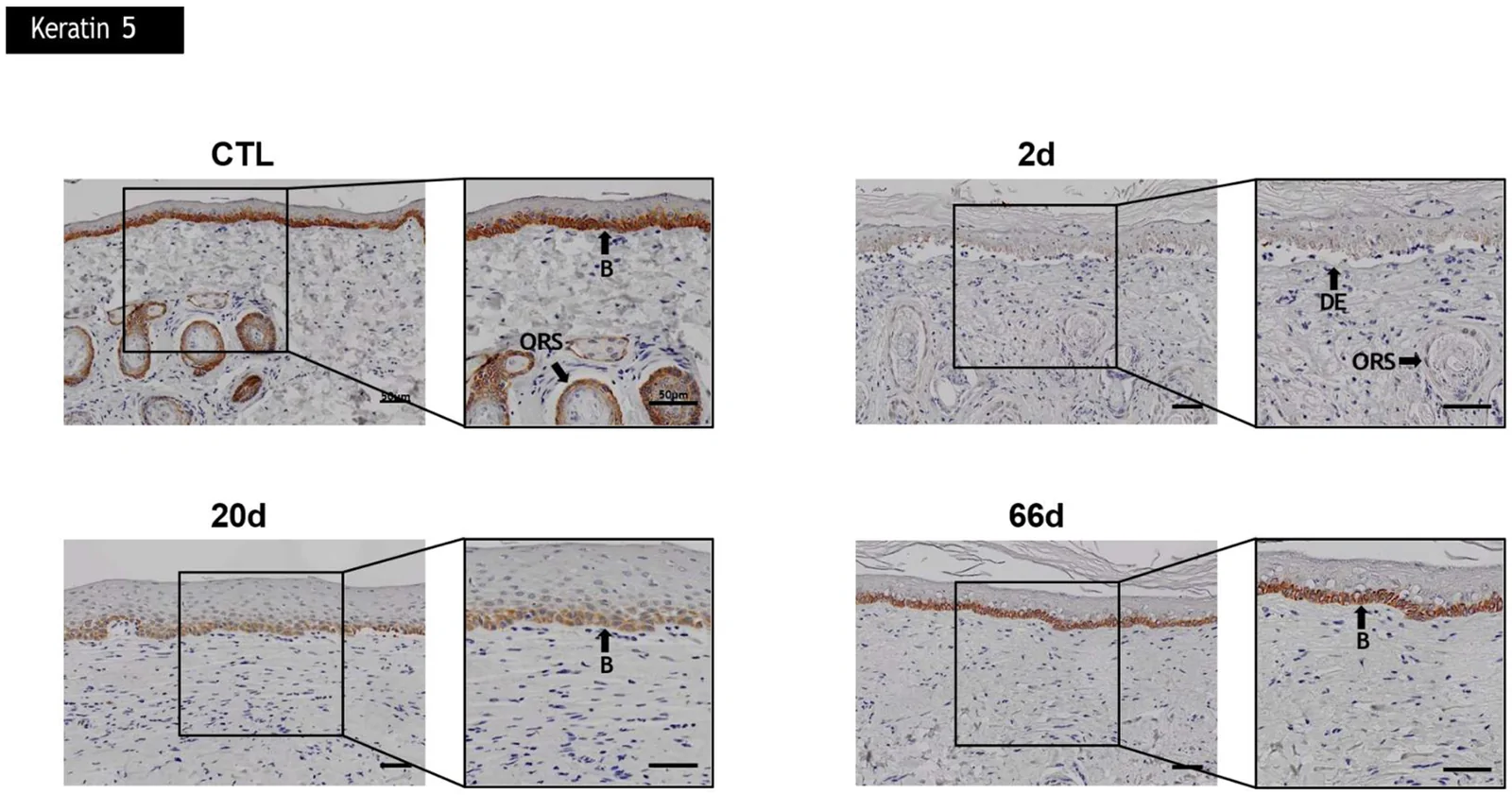
Localization of keratin 5 in hairless guinea pig skin following SM exposure. Control (CTL) guinea pig skin and 2-, 20-, 66-days post-SM guinea pig skin sections were stained with antibodies against keratin 5. Antibody binding was visualized using a Vectastain Elite ABC kit. Images at low and high magnification are presented. B, basal layer; DE, dermo-epidermal cleft; ORS, outer root sheath of the hair follicle. Original magnification, x200.

**Figure 9:**
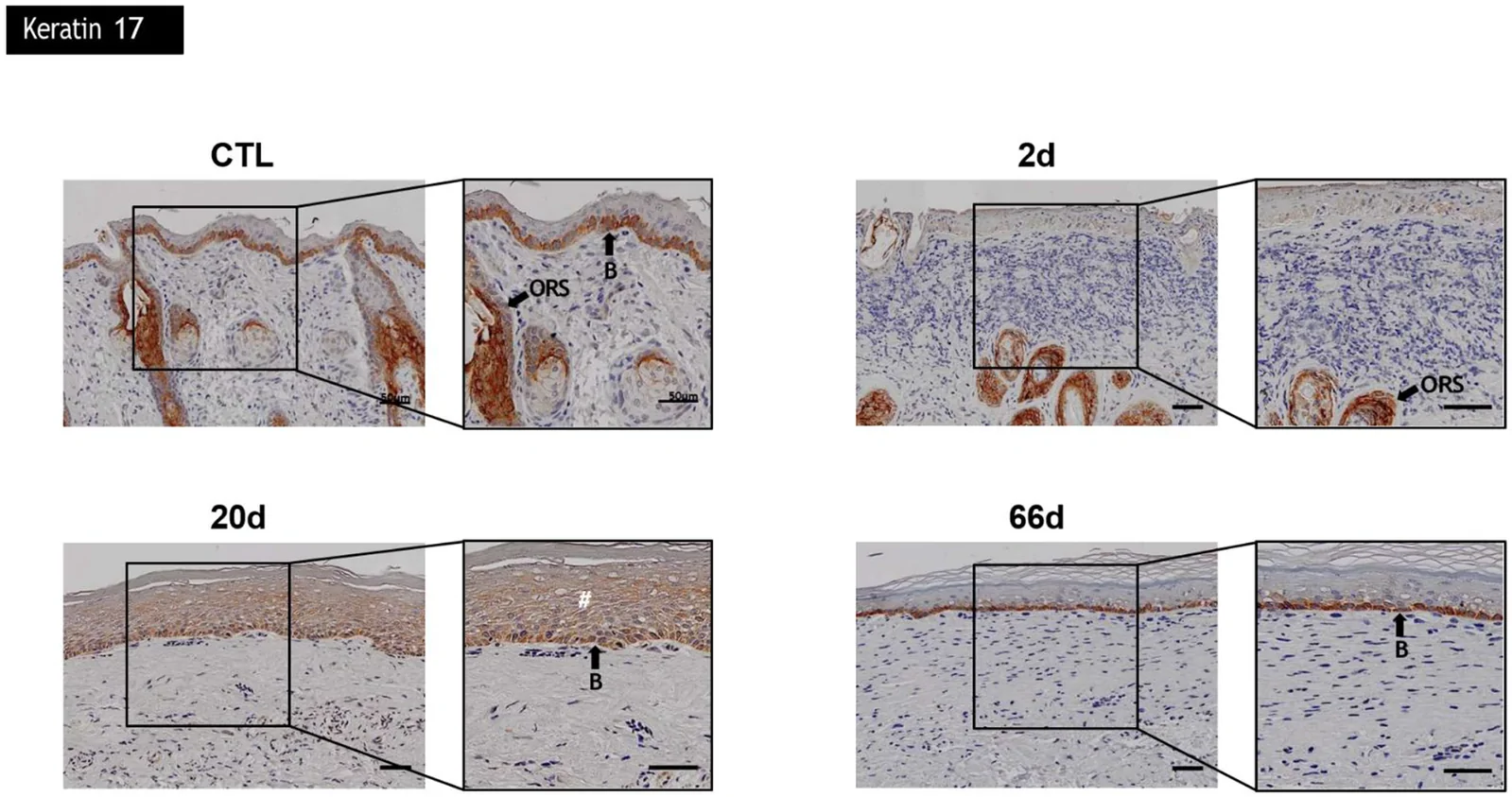
Localization of keratin 17 in hairless guinea pig skin following SM exposure. Control (CTL) guinea pig skin and 2-, 20-, 66-days post-SM guinea pig skin sections were stained with antibodies against keratin 17. Antibody binding was visualized using a Vectastain Elite ABC kit. Images at low and high magnification are presented. B, basal layer; ORS, outer root sheath of the hair follicle; Pound sign, suprabasal layer. Original magnification, x200.

**Figure 10:**
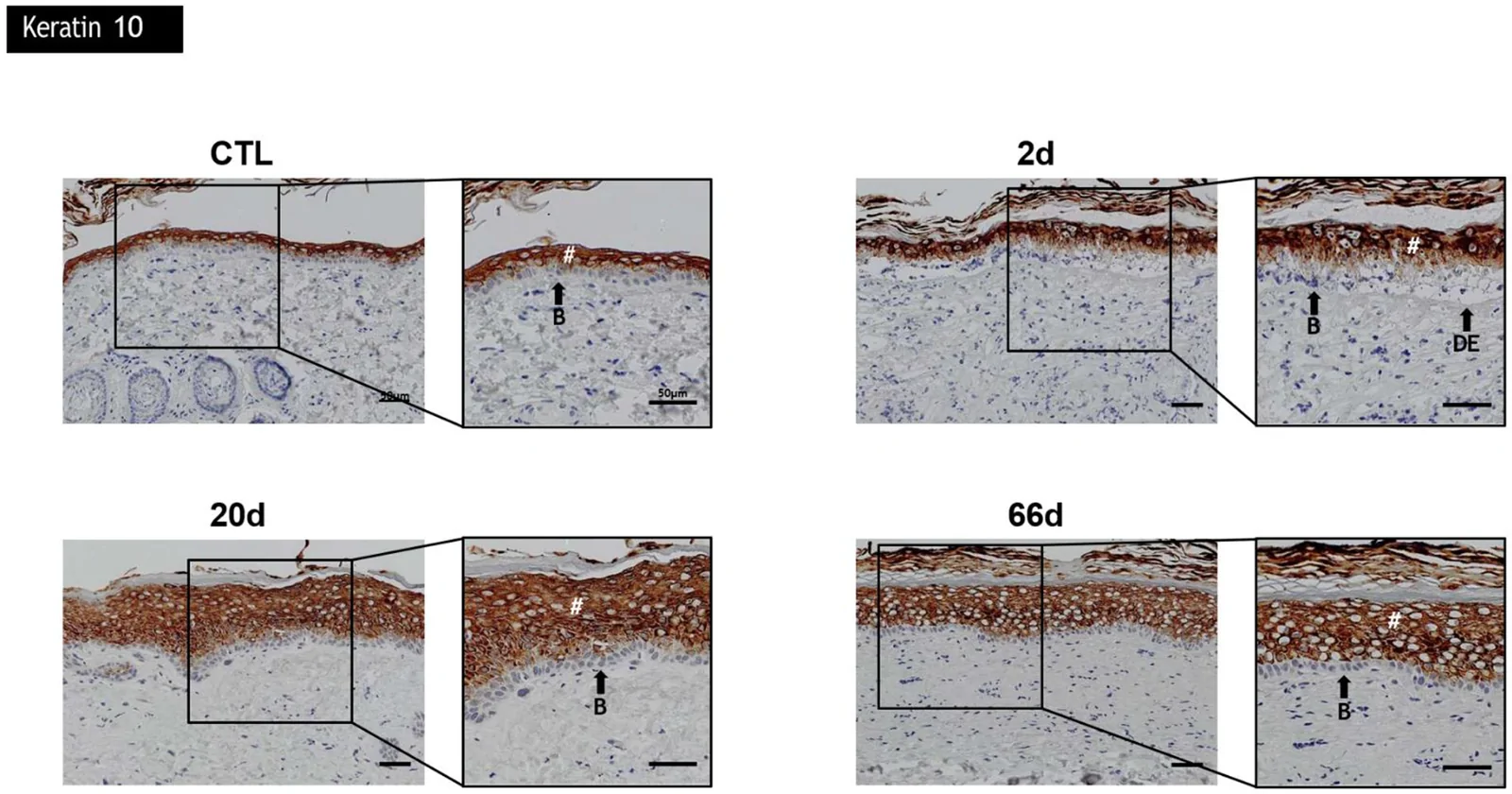
Localization of keratin 10 in hairless guinea pig skin following SM exposure. Control (CTL) guinea pig skin and 2-, 20-, 66-days post-SM guinea pig skin sections were stained with antibodies against keratin 10. Antibody binding was visualized using a Vectastain Elite ABC kit. Images at low and high magnification are presented. B, basal layer; DE, dermo-epidermal cleft; Pound sign, suprabasal layer. Original magnification, x200.

**Figure 11:**
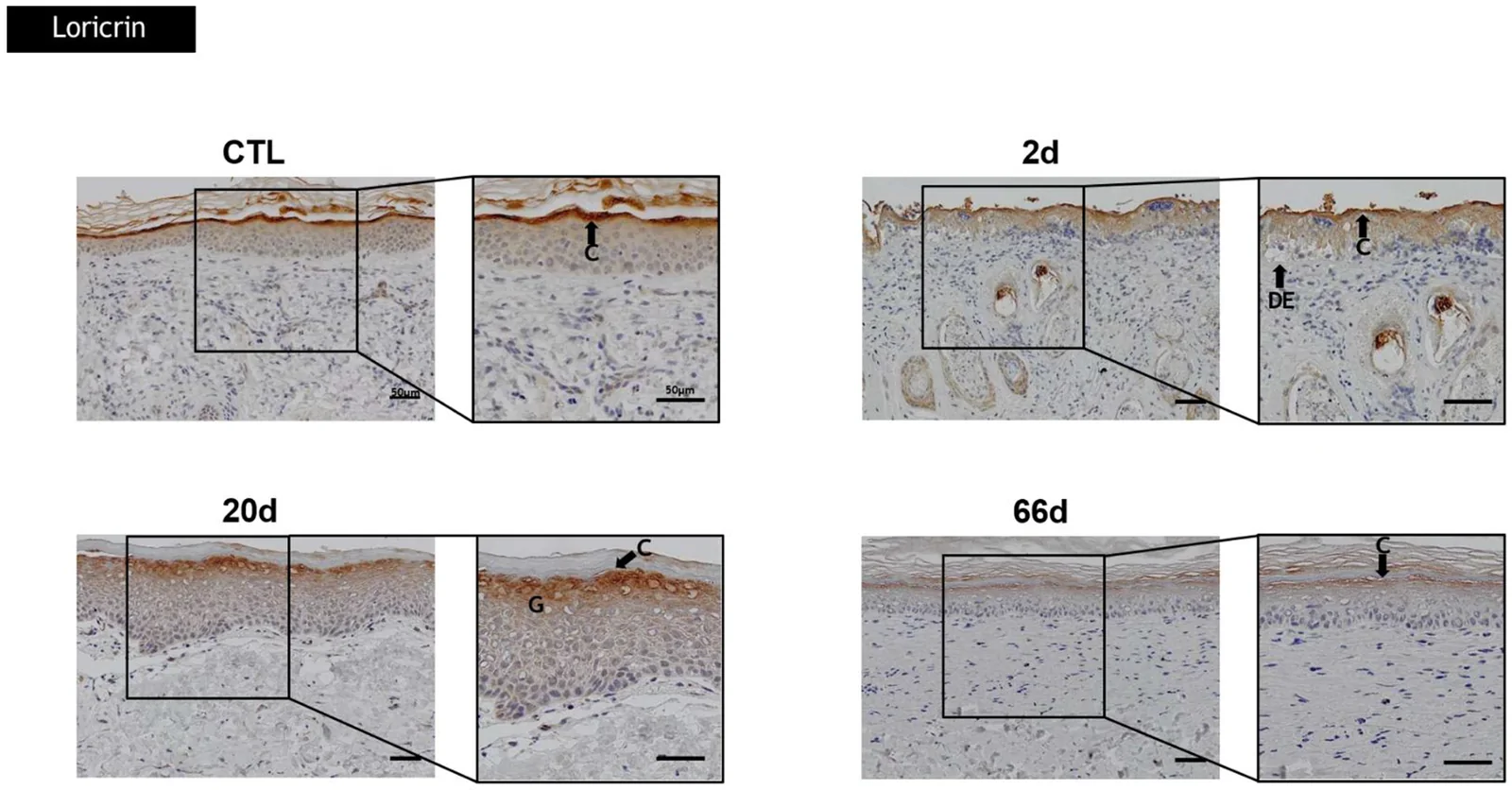
Localization of loricrin in hairless guinea pig skin following SM exposure. Control (CTL) guinea pig skin and 2-, 20-, 66-days post-SM guinea pig skin sections were stained with antibodies against loricrin. Antibody binding was visualized using a Vectastain Elite ABC kit. Images at low and high magnification are presented. C, cornified envelope; DE, dermo-epidermal cleft; G, granular layer. Original magnification, x200.

## Data Availability

The data supporting the findings of this study are available from the corresponding author upon reasonable request.

## Abbreviations

DEJ: Dermal-Epidermal Junction
PBS: Phosphate-Buffered Saline
PCNA: Proliferating Cell Nuclear Antigen
SM: Sulfur Mustard
